# Effect of symbiont-targeted control of *Halyomorpha halys* on the co-occurring pentatomid community

**DOI:** 10.3389/finsc.2025.1520065

**Published:** 2025-02-05

**Authors:** Sofia V. Prieto, Bianca Orrù, Elena Gonella, Alberto Alma

**Affiliations:** Department of Agricultural, Forest and Food Sciences, University of Torino, Grugliasco, Italy

**Keywords:** symbiosis disruption, Pentatomidae, *Pantoea*, V4 gut ventricle, secondary pest, non-target organisms

## Abstract

Several native species in the family Pentatomidae are recorded in north-western Italy, associated with different crops. The arrival of *Halyomorpha halys* led to a reorganization of the role of other pentatomids, some of them becoming secondary pests. Symbiont-targeted control strategies, which disrupt beneficial interactions in stink bugs, have so far been applied to *H. halys*. However, this approach could also be useful for controlling other pentatomid pests. Additionally, the effects of this strategy on non-target stink bug species need further investigation to assess its potential impact on agroecosystems. Here the effect of symbiont disruption was assessed for stink bugs that share host crops (*e.g.*, hazelnut, wheat, soybean) or the environment (especially wild areas adjacent to crops) with *H. halys* in north-western Italy (*Carpocoris purpureipennis*, *Dolycoris baccarum*, *Graphosoma italicum*, *Palomena prasina* and *Rhaphigaster nebulosa*). Their symbionts were identified as allied to the genus *Pantoea* through 16S rRNA gene sequencing and also other bacteria were detected in the V4 ventricle of the midgut. Strikingly, variable symbiont infection was found across species. Laboratory tests were conducted assessing the consequences of symbiont deprivation during the first nymphal instar. Egg masses treatment with an anti-symbiont formulation affected hatching rates in *D. baccarum* and *G. italicum*, while the mortality rates during the first instar increased in *C. purpureipennis* and *G. italicum*. A correspondence between mortality induction and the alteration of symbiont infection rates was observed, with species showing the highest infection drop being the most affected by treatments. These results provide new insights into pentatomid symbionts and reveal significant variability in the response to symbiosis disruption, likely due to species-specific intensity of symbiotic interactions. The consequences of this variability are discussed.

## 1 Introduction

Pentatomid pests are responsible for considerable economic losses in different crop productions in north-western (NW) Italy (1, 2). Stink bugs mouthparts are needle-shaped and allow the insect to penetrate the vegetal tissue and insert digestive enzymes that contribute to its nourishment, while causing direct and indirect damage to the fruit (*e.g.*, premature abscission, fruit deformation and/or weight reduction, entrance of pathogens) (3). The polyphagous feeding behaviour of stink bugs allow them to feed on different plant families, both cultivated and not (3); this last determines their presence also in the surrounding areas of crops compromising the efficacy of chemical treatments in-field (4). Usually, the community of pentatomid pests associated with a crop consists of one or two key species, causing the highest damages, and other secondary species representing a variable degree of hazard (5). In some cases, agricultural management decisions can lead to a decrease in a key species and a subsequent boost of a secondary one, due to the cessation of conditions that had previously regulated that species, such as suppression of natural enemies through insecticide treatments (6). For example, in Argentina and Brazil the no-tillage cultivation system spread in the last decades has led (among other factors) to a reduction in *Nezara viridula* (Linnaeus) population associated with the soybean crop, followed by a rise of stink bugs populations so far considered secondary pests such as *Dichelops furcatus* (Fabricius) and *Euschistus heros* (Fabricius) (7). In this context, secondary pests can become key species causing high damage (8). The integrated pest management requires a holistic approach when choosing a control strategy, which considers not only the key pest but also the impact on the associated species.

In hazelnut orchards in Europe (*i.e.*, NW Italy) and Turkey, indigenous pentatomid species such as *N. viridula* and *Palomena prasina* (Linneaus), along with coreid species like *Gonocerus acuteangulatus* (Goeze), were traditionally considered key pests. However, the recent invasion of the exotic species *Halyomorpha halys* Stål has altered this dynamic (1). The fast expansion of *H. halys* upon arrival can be attributed at least partially to its highly polyphagous habit and reproduction capacity, and to the absence of relative efficient natural enemies (9, 10). However, species such as *P. prasina* are still causing huge damage to the hazelnut production in Turkey, so the impact of secondary species should not be underestimated (11).

Another crop that is threatened by heteropteran pests in NW Italy is wheat, where infestations by the so-called Sunn pests, belonging to the genera *Eurygaster* (Hemiptera: Scutelleridae) and *Aelia* (Hemiptera: Pentatomidae) are usually recorded together with secondary pests such as *Carpocoris purpureipennis* (De Geer). Although lower infestation levels of *C. purpureipennis* are usually detected in wheat, according to Vaccino et al. (2) this species is almost as noxious as *E. maura* regarding the capacity of damage induction. In the surrounding areas of wheat crops and hazelnut orchards in NW Italy, as well as in soybean fields, also the stink bug *Dolycoris baccarum* (Linneaus) is often present, mainly feeding on secondary hosts. This species is considered a notorious pest for diverse crops in the United States, such as sunflower, tobacco and cereal grains (12, 13), and in Japan, attacking soybean, cotton, sesame, carrots and rice (14). Other pentatomid species integrating the community of stink bugs present in NW Italy include *Graphosoma italicum* (Müller), very often in strict association with Apiaceae plants. *Graphosoma italicum* is hardly ever considered a dangerous pest in agriculture, so as *Rhaphigaster nebulosa* (Poda), another pentatomid commonly observed in NW Italy during autumn, when it searches for an overwintering location, usually inside the buildings (15).

In recent years, biological control strategies using hymenopteran egg parasitoids, primarily from the Scelionidae family, have been increasingly used in NW Italy and elsewhere to control pentatomid pests (16). In particular, the egg parasitoid *Trissolcus japonicus* (Ashmead) has proven to be very effective and is currently incorporated in a number of control programs targeting *H. halys* (17). The arrival and fast spread of *H. halys* in Europe, however, has promoted the search of other complementary strategies. Unravelling insect beneficial interactions (*i.e.*, mutualistic symbioses) with bacteria can be crucial in new pest management approaches, considering the key role played by bacteria in the life of the host in a variety of aspects from nutrient provision to immunity (18). In stink bugs, gut symbionts provide essential nutrients to the host, such as amino acids and vitamins; altering the synergy with symbiotic bacteria usually compromises the insect fitness (19). In this context, disrupting this symbiosis becomes the essence of the symbiotic control as a pest management strategy conducted through symbiont-targeted treatments. This approach aims at interrupting insect acquisition of beneficial symbiotic bacteria (19). In pentatomid insects, symbiont transmission happens vertically by egg smearing, meaning that the females release a symbiont-containing secretion on the egg surface during oviposition, allowing for the subsequent symbiont acquisition by the newly hatched nymphs (20). The symbiotic control involves treating the egg mass with anti-symbiont compounds in order to block symbiont acquisition (21, 22). This strategy has proven to be effective in the laboratory against *H. halys*, whilst a variable effect was observed for other species (23–25).

To fully understand the effectiveness and limitations of symbiotic control, this strategy must be tested not only on target species but also on secondary pests and non-pest species. Duron and Noël (26) conducted a multilocus phylogenetic study that classified the symbionts of *C. purpureipennis*, *D. baccarum* and *G. italicum* within the genus *Pantoea*. The symbiont of *R. nebulosa*, on the contrary, fell off this genus. In addition, they evaluated the effect of symbiont elimination on the mortality of *D. baccarum* and *G. italicum*, indicating a detrimental effect on the former and no effect on the second. Similar results were obtained by Itoh et al. (27) on *D. baccarum*. On the contrary, a negative effect was observed on *G. lineatum* (closely related to *G. italicum*) after symbiont depletion by Karamipour et al. (28). Although *P. prasina* is an important pest in hazelnut orchards, to our knowledge, no studies have investigated the effects of symbiont elimination in this species.

The aim of this study was to assess if the stink bug community occurring in an area where symbiont-targeted control of *H. halys* is often conducted, using a commercial biocomplex with antimicrobial effect, is affected by treatments inducing a symbiosis disruption. To evaluate species-specific sensitivity to symbiotic control, we firstly identified the primary symbiont of the most common stink bugs collected in different crops, subsequently anti-symbiont treatment was performed on their egg masses under laboratory conditions, and the effect of disrupting the symbiotic interaction on nymph fitness was evaluated.

## 2 Materials and methods

### 2.1 Collection and laboratory rearing of stink bugs

Adults of all tested species were collected during 2022 in different locations of Piedmont, NW Italy (**Supplementary Table S1**). The following species were found in field during the spring-summer season: *P. prasina* specimens were collected in a hazelnut orchard; individuals of *C. purpureipennis* were found in a wheat field; *D. baccarum* individuals were collected in a soybean field; *G. italicum* adults were collected from spontaneous *Anthriscus sylvestris* (Apiaceae), a common host plant. Specimens of *R. nebulosa* were collected from *Acer* spp. trees during the autumn of 2022. Separate insect colonies were established in mesh cages (930 × 475 × 475 mm), in a climatic room at 25°C and 70% RH and under a long-day regimen (16 h light, 8 h dark). All species were fed on hazelnuts, apples and fresh beans, except from *G. italicum*, provided with fresh *A. sylvestris* as feeding source and oviposition surface. In *P. prasina* cages, a *Vicia faba* (Fabaceae) plant was collocated to facilitate oviposition, while *C. purpureipennis* and *D. baccarum* were provided with plants of *Avena sativa* (Poaceae). Cages were checked daily, and egg masses were collected and used for further experiments.

### 2.2 Stink bug gut dissection for symbiont characterization

A total of 20 adults per species were immersed in 70% ethanol for 3 minutes to surface sterilize them before being dissected for midgut analysis using fine forceps. Dissections were performed inside a Petri dish with 500μl of a sterile NaCl solution 0.9%, under a dissection microscope. The V4 ventricle of the midgut, recognisable by its four tubular outgrowths, was separated from the rest of the midgut and kept in single 1.5mL Eppendorf tubes at -20°C for DNA extraction.

### 2.3 DNA extraction, 16S rRNA gene PCR and sequencing

The V4 gut section of stink bugs was subject to DNA extraction using a Phenol Chloroform protocol (29). The quality of the extracted DNA was checked using the Thermo Scientific NanoDrop™ 1000 Spectrophotometer (Thermo Fisher Scientific^®^) and further confirmed by agarose gel electrophoresis. Considering that the V4 region of pentatomids is expected to be dominated by a single symbiont (26), symbiont identification was performed in all 20 samples of each species by amplicon Sanger sequencing. PCR targeting the bacterial 16S rRNA gene was conducted, initially with universal primers 357f-/907r-, expecting ~550 bp fragments for a first screening of bacterial presence, and afterwards with universal primers 16Sd-/16Sr- aiming to obtain larger fragments (~1200 bp) for a more accurate symbiont phylogenetic reconstruction. The sequences of all primers used in this study, along with their annealing temperature profiles and references are detailed in **Table 1**. Polymerase chain reaction was conducted with HOT FIREPol^®^ DNA polymerase (Biosigma). After visualization of PCR products through electrophoresis in 1% agarose gel, positive samples were purified with the QIAquick^®^ PCR purification Kit (Qiagen) and submitted to Sanger sequencing (Eurofins Genomics, Germany). Sequences were afterwards manually checked and trimmed according to the quality scores using software Chromas version 2.6.6. Sequences were confronted with the GenBank database of the NCBI (https://www.ncbi.nlm.nih.gov/genbank), using the tool BLAST (30). Symbiont 16S rRNA gene sequences were deposited in the GenBank database under accession numbers PP469563 to PP469568.

**Table 1:** List of primers used in this study for insect and symbiont identification.

Gene	Primers (5’-3’)		Annealing temperature and time	Fragment size (bp)	Reference
	Name	Oligonucleotide			
insect COI	LCO-	GGTCAACAAATCATAAAGATATTGG	50°C, 45’’	710	(47)
	HCO-	TAAACTTCAGGGTGACCAAAAAATCA			
16S rRNA (universal)	16Sd-	GCTGGCGGCATGCTTAACACAT	50°C 45’’, (×5) + 55°C, 45’’, (×30)	1200	(48)
	16Sr-	GGAGGTGATCCAGCCGCAGGT			
	357f-	CTACGGGAGGCAGCAGT		550	(49)
	907r-	CCGTCAATTCCTTTGAGTTT			
16S rRNA (specific)					
Symbiont of *Carpocoris purpurepennis*	Pcarp1F-	GATGACCAGCCACACTGGAA	54°C, 45’’	165	this study
	Pcarp1R-	TTAACCACGTCGCCTTCCTC			
Symbiont of *Dolycoris baccarum*	BCHGf1-	CAGATGGGATTAGCCAGTAG	55°C, 45’’	470	(27)
	BCHGr1-	CTACACATTTCACCGCTACA			
Symbiont of *Graphosoma italicum*	Pgra1F-	CCAGAGCACTTGGCAGAGAT	54°C, 45’’	167	this study
	Pgra1R-	CCGGCAGTCTCCTTTGAGTT			
Symbiont of *Palomena prasina*	PPp1F-	AGAGATGGCTTGGTGCCTTC	55°C, 45’’	150	this study
	PPp1R-	GCAGTCTCCTTTGAGTCCCC			

Stink bug species were identified after collection by direct observation of morphological characters. For *C. purpureipennis* and *G. italicum* the taxonomical assignment was confirmed by amplifying the insect cytochrome oxidase subunit I gene (COI) in three randomly chosen individuals, with primers LCO/HCO (**Table 1**), using the V4 ventricle DNA as template. Amplicons were sequenced as detailed above and stink bug species was confirmed according to COI sequences first match on the NCBI database (highest Percent Identity). The obtained COI gene sequences of *C. purpureipennis* and *G. italicum* are available in the GenBank database under accession numbers PP469574 and PP469575, respectively.

### 2.4 Molecular phylogenetic analysis of primary stink bugs symbionts

A Maximum-likelihood (ML) approach was chosen to analyse the phylogenetic placement of the symbionts, on the basis of the obtained 16S rRNA gene sequences and a selection of sequences, namely: 1) from the NCBI database, taxonomically assigned to pentatomid symbionts; 2) from Erwiniaceae bacteria, mainly *Pantoea* and *Erwinia*, recorded in nymphs of *N. viridula* by Prieto et al. (24); and 3) a strain of *Yersinia pestis* that was used as an outgroup. The sequences were aligned using the MUSCLE algorithm (31). A pairwise distance analysis was performed to eliminate potential equal sequences from further analysis. Tamura 3-parameter substitution model with Gamma distribution and proportion of invariant sites was selected as best nucleotide substitution model. Maximum-Likelihood gene tree was inferred from 1080 aligned nucleotide sites using MEGA-X software (32) (selected option: gap treatment = partial deletion; bootstrap = 2,000 replicates; tree searching method = *Nearest-Neighbor-Interchange*).

### 2.5 Symbiont-targeted experiments on egg masses and measurement of first instar mortality

Egg masses of each tested species (*C. purpureipennis*, *D. baccarum*, *G. italicum* and *P. prasina*) were daily collected and the total number of eggs per egg mass was recorded. Subsequently, egg masses were randomly assigned to one treatment: sprayed with an anti-symbiont formulate (the micronutrient biocomplex Dentamet^®^, Diachem S.p.a., Italy) previously used to eliminate symbiotic bacteria from the egg surface of other stink bugs (22, 24), or untreated control (same conditions as the experimental group by without any symbiont disruption treatment), as detailed in Gonella et al. (22). The final number of replicates varied for each species based on the availability of egg masses, with a minimum number of replicates per treatment equal to ten. For each tested species, the number of eggs per egg mass did not change between treatments (unpaired Student’s *t* test, n.s.). After the treatment, egg masses were kept in the same climatic room where the stink bugs colonies were reared and under equal environmental conditions. For each replicate, the hatching date, the number of hatched eggs and the total number of nymphs reaching the second instar were recorded. These values were used afterwards to calculate the time between egg laying and egg hatching, the hatching rate and the mortality rate during the first instar. Once nymphs either died or reached the second instar the experiment was ended.

### 2.6 Diagnostic symbiont-specific PCR on nymphs derived from egg masses experiments

To confirm the disruption of symbiont acquisition by anti-symbiont treatment of egg masses, 20 second instar nymphs of all species tested in symbiont-targeted experiments, ten per category (derived from egg masses treated with the anti-symbiont biocomplex and from the untreated control) were subject to RNA extraction and reverse transcription following the protocol used by Gonella et al. (22). Nymphs for molecular analysis were randomly selected from egg masses of each category; no more than three nymphs were collected from the same egg mass. The cDNA was used as a template for end-point PCR reactions targeting the 16S rRNA gene of each symbiont (including all recorded variants) with specific primers designed in this study, except for the symbiont of *D. baccarum* whose specific primers were obtained from Itoh et al. (27) (**Table 1**). Primers were designed using NCBI Primer-BLAST, based on the symbiont 16S rRNA gene sequences obtained in this study (**Table 1**). Primers were tested in silico using software SerialCloner.

### 2.7 Statistical analysis

Hatching and mortality rates were statistically assessed by means of a Generalized Linear Model (GLM) with binomial probability distribution and logit link function. Egg masses maturation time was evaluated using the Kruskal-Wallis test. Symbiont presence in nymphs after egg masses experiments was assessed using the Fisher’s Exact test. All statistical analysis and graphical procedures were performed using software R (33).

## 3 Results

### 3.1 Molecular identification of *C. purpureipennis* and *G. italicum*

The COI gene sequences, obtained from the stink bug for which a molecular identification was needed, were approximately 640 bp long. Comparing these sequences within the GenBank database, all three sequences of *C. purpureipennis* and *G. italicum* matched with sequences assigned to those species, respectively, confirming correct species identification (first match accession number JN871531 and KX960063, respectively).

### 3.2 Variability of symbiotic and non-symbiotic bacteria strains recorded in the V4 ventricle of stink bugs

The DNA extracted from the V4 ventricle was screened with primers 357f-/907r, obtaining fragments of about 530 bp (500 – 550 bp). PCR confirmed the presence of bacteria in the V4 ventricle in all species; however, when confronting the obtained sequences with the NCBI database, some amplicons matched with species out of the genus *Pantoea*, which is the one expected to include primary symbionts of Pentatomidae (26). More specifically, all sequences obtained from *G. italicum* and *R. nebulosa* matched with *Pantoea* sequences that have been previously reported as their primary symbionts (top hits between 98 and 100% similarity). In contrast, only 78% of sequences from *D. baccarum* (11 out of 18 sequences), 40% from *P. prasina* (4 out of 10 sequences), and 8% from *C. purpureipennis* (1 out of 12 sequences) were assigned to *Pantoea* symbionts. Symbionts of *D. baccarum* and *C. purpureipennis* matched with sequences that have been previously reported as the primary symbiont of these species, whereas sequences from *P. prasina* were strictly related to the primary symbiont of *Palomena angulosa* (Motschulsky), with a 99% similarity (query accession number of the first match LC168541). The other sequences corresponded to different bacteria, mainly proteobacteria. The four sequences that were not assigned to the primary symbiont detected in *D. baccarum* samples corresponded to *Cedecea* spp., *Pantoea agglomerans*, *Serratia marcescens* and an undefined Enterobacteriaceae. For *P. prasina*, an undefined bacterium in the Acetobacteraceae family was recorded in three samples, while the remaining three non-*Pantoea* symbionts belonged to the genus *Serratia* (two out of three sequences being assigned to *S. marcescens*). In samples of *C. purpureipennis*, a higher level of variability was observed, as 11 out of 12 sequences did not correspond to the *Pantoea* symbiont, but rather to *Enterococcus faecalis* (four sequences), *Serratia* (two sequences corresponding to *Serratia* spp. and two to *S. marcescens*), *Enterococcus* sp. (one sequence), *Yokenella regensburgei* (one sequence) and an undefined Acetobacteriaceae (one sequence).

### 3.3 Phylogenetic placement of strains assigned to stink bugs symbionts

**Figure 1:**
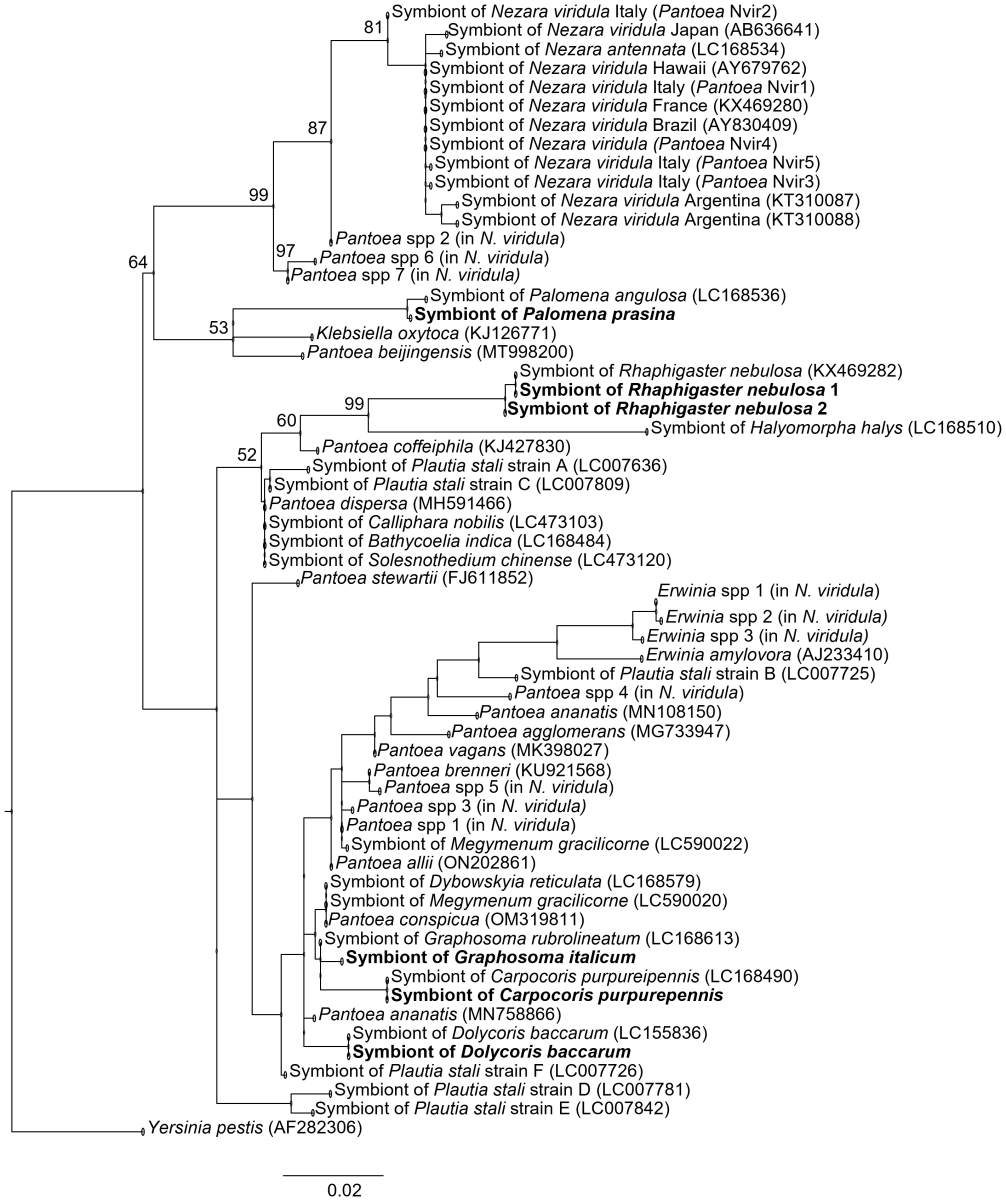
Phylogenetic placement of gut symbionts of *Carpocoris purpureipennis, Dolycoris baccarum, Graphosoma italicum, Palomena prasina* and *Rhaphigaster nebulosa* collected in Piedmont (Italy), based on 16S rRNA gene sequencing. Sequences obtained in this study are written in bold. Round brackets indicate the accession numbers. Branch numbers are the percentage bootstrap support values for major branches (2,000 bootstrap replicates; only bootstrap values higher than 50% are shown). The scale bar indicates units of substitution per site.

Results on the phylogenetic placement of the stink bugs symbiotic bacteria based on the 16S rRNA gene are shown in **Figure 1**. For phylogenetic analysis, the ~1200 bp sequences amplified with universal primers 16Sd-/16Sr- were used. No different symbiont variants were observed for *D. baccarum*, *G. italicum* and *P. prasina* according to pairwise distance analysis. Conversely, two variants were recorded for the symbiont of *R. nebulosa*, each variant represented by nine and two samples, respectively. No variability assessment was possible for the symbiont of *C. purpureipennis* since only one sequence was obtained.

The phylogenetic tree shows that the described symbionts are distributed in two clades. The first (upper) clade is separated in two subclades, one of them containing the symbiont of *P. prasina*, which clusters together with the symbiont of the related species *P. angulosa*. The other sub-clade is composed by *N. viridula* symbionts from different geographical areas. The symbiont sequences from all the tested stink bugs other than *P. prasina* fell into the second clade, although separated in two subclades: the symbionts of *C. purpureipennis*, *D. baccarum* and *G. italicum* were grouped together in the lower subclade, along with *Pantoea* and *Erwinia* strains and other stink bugs symbionts. The symbiont of *G. italicum* was closer phylogenetically to the symbiont of *Graphosoma rubrolineatum* (Westwood), while the sequences of the symbionts of *C. purpureipennis* and *D. baccarum* were equal to sequences present in the NCBI database and previously assigned to the same species. Finally, the two variants assigned to *R. nebulosa* symbiont were allocated in the other subclade: variant 1 was equal to a deposited sequence of *R. nebulosa* symbiont, while variant 2 clustered separately, showing slight divergency from sequence 1 (99.7% similarity).

### 3.4 Hatching and first instar mortality rates after anti-symbiont treatment of egg masses

Fresh egg masses of *C. purpureipennis, D. baccarum, G. italicum* and *P. prasina* were collected from the laboratory colonies and sprayed with an anti-symbiont biocomplex. In contrast, no egg masses were obtained from our *R. nebulosa* colony due to difficulties in mass rearing of this species, so the latter was not included in the symbiont disruption experiments. The time between collection of the egg mass and egg hatching was the same for all species except for *P. prasina*, whose eggs underwent a faster hatching when sprayed with the anti-symbiont biocomplex (Treated: 3.7 ± 1.3 days, *n*=12; Control: 5.0 ± 1.3 days, *n*=10; Kruskal-Wallis test, *P *= 0.021; **Table 2**).

**Table 2:** Number of replicates, number of eggs per egg mass, total of first and second instar nymphs, hatching rates and first instar mortality rates for each species according to the treatment (untreated control and the commercial biocomplex Dentamet^®^).

Species	Treatment	Replicates (n)	Eggs per egg mass (n)	First instar nymphs (n)	Hatching (%)	χ2	*P* value	Second instar nymphs (n)	Mortality first instar (%)	χ2	*P* value
*Carpocoris purpureipennis*	Control	25	14.6 ± 6.8	3.8 ± 1.8	26 ± 5.8	2.37	0.125 (n.s.)	3.4 ± 1.7	49.8 ± 12.6	62.34	< 0.000 (***)
	Dentamet^®^	25	15.8 ± 7.0	4.8 ± 2.1	28.8 ± 6.2			0.7 ± 0.6	82.2 ± 7.7		
*Dolycoris baccarum*	Control	10	17.5 ± 7.0	11.7 ± 5.1	68.9 ± 8.7	24.51	< 0.000 (***)	8.7 ± 3.6	34.1 ± 11.8	0.51	0.475 (n.s.)
	Dentamet^®^	10	23.6 ± 7.6	16.7 ± 4.4	36.1 ± 12.2			11.7 ± 2.8	53.4 ± 17.8		
*Graphosoma italicum*	Control	19	13.6 ± 6.9	8.2 ± 3.4	37.2 ± 8.9	77.67	< 0.000 (***)	5.9 ± 2.6	36.6 ± 11.7	11.8	< 0.001 (***)
	Dentamet^®^	19	14.0 ± 8.3	11.4 ± 5.2	75.7 ± 8.7			6.5 ± 2.9	53.1 ± 8.1		
*Palomena prasina*	Control	12	11.8 ± 5.9	6.9 ± 2.8	58.4 ± 10.7	0.55	0.459 (n.s.)	3.4 ± 1.5	60.1 ± 13.4	0.1	0.752 (n.s.)
	Dentamet^®^	12	16.4 ± 5.9	10.3 ± 3.9	60.0 ± 8.8			4.8 ± 2.0	52.2 ± 12.9		

For hatching and mortality rates, the Chi Square value and the *P* value with its associated significance are shown, according to binomial GLM. Results are expressed as mean values ± SEM. *** indicates P value lower than 0.001. n.s. means no significant differences were detected.

No differences related to treatment were observed in the hatching rates of egg masses of *P. prasina* and *C. purpureipennis* (**Table 2**). Contrastive results were observed, on the contrary, for *D. baccarum* and *G. italicum*: in the case of *D. baccarum*, hatching rates decreased significantly in egg masses after anti-symbiont treatment; the opposite effect was observed for *G. italicum*, which expressed significant higher hatching rates in treated egg masses (**Table 2**).

**Figure 2:**
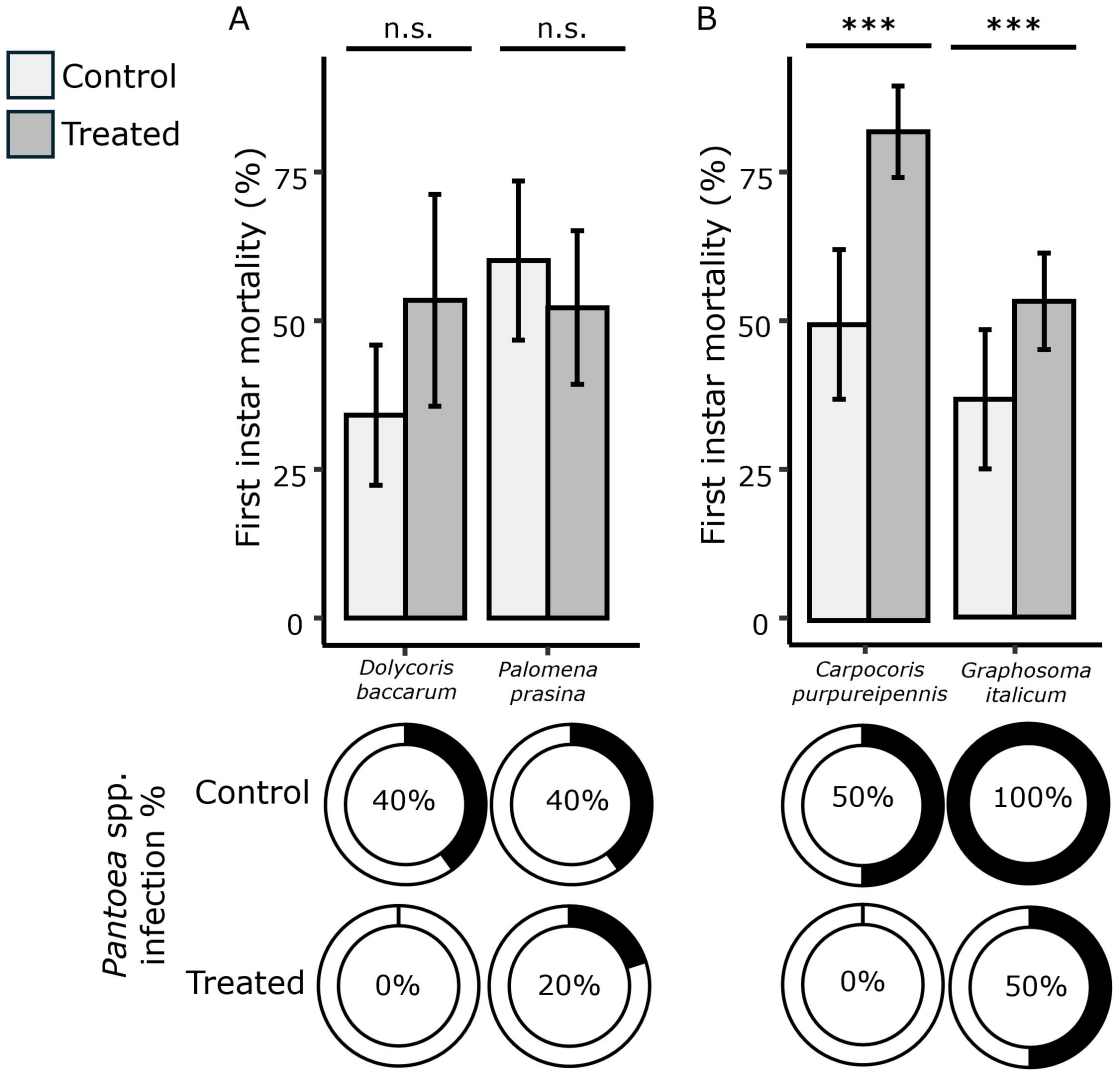
Effect of anti-symbiont treatment on egg masses of *Carpocoris purpureipennis, Dolycoris baccarum, Graphosoma italicum*, and *Palomena prasina.* First instar mortality rates are indicated along with symbiont infection rates, as recorded through PCR with specific primers performed on second instar nymphs of the tested stink bugs, namely *D. baccarum* and *P. prasina* **(A)**, and *C. purpureipennis* and *D. baccarum* **(B)** (white bars, control; grey bars, anti-symbiont treatment). Error bars indicate mean standard error. Asterisks (***) indicate significance differences between adjacent bars according to binomial GLM analysis (*P* < 0.001). n.s., not significant.

Concerning first instar mortality rates, no significant differences were observed for *D. baccarum* and *P. prasina* when comparing nymphs derived from egg masses treated with the biocomplex and untreated samples (**Figure 2A**, **Table 2**). On the contrary, the first instar mortality increased significantly in nymphs from the species *C. purpureipennis* and *G. italicum* (**Figure 2B**, **Table 2**).

To confirm symbiont absence after treatment with the anti-symbiont biocomplex, the cDNA of second instar nymphs from all species and treatment categories was submitted to PCR with symbiont-specific primers (**Figure 2**). Significant differences were observed in the number of symbiont-positive specimens when comparing nymphs derived from egg masses treated with the biocomplex against the untreated controls for *C. purpureipennis* and *G. italicum* (Fisher’s exact test: *C. pupureipennis*: *P* = 0.032. *G. italicum*, *P* = 0.032), whose natural infection in the absence of treatment was the highest (50-100%). Conversely, despite lower symbiont incidence was found after the treatment also in *D. baccarum* and *P. prasina*, no significant differences were observed in this case, because in these species also natural infection without treatment was low, not surpassing 40%.

## 4 Discussion

When evaluating a novel pest control technique, it is crucial to assess its impact on the insect communities that may be exposed to it. In this context, this work has examined several pentatomid species that are usually recorded in the Piedmont region of NW Italy. A first outcome of this study was the identification of *Pantoea* symbionts residing in the V4 midgut ventricle of the studied stink bugs. This region is specialized for housing symbiotic bacteria, and becomes morphologically isolated from the anterior midgut parts during the insect development after symbionts are orally acquired; thanks to such high specialization it is easily recognizable by its four tubular outgrowths (19, 20, 34). We detected at least one sequence referable to the *Pantoea* symbiont in all tested species. The phylogenetic positioning of the symbionts is mostly in agreement with previous works that addressed the phylogenetic aspects of the studied stink bugs symbionts (26, 27), as we confirmed that symbionts of *C. purpureipennis*, *G. italicum* and *D. baccarum* clustered together and separately from the symbiont of *R. nebulosa*. According to our study, all symbiont sequences appeared to be placed in the genus *Pantoea*. However, according to an MLST approach reported by Duron and Noël (26), the symbiont of *R. nebulosa* fell outside this genus; therefore, our results regarding the phylogenetic position of this strain are not conclusive and could be reinforced by testing other genes apart from the 16S rRNA. This may additionally support investigations on the symbiont of *C. purpureipennis*, for which we failed at obtaining more than one 16S rRNA gene sequence, possibly due to a degree of variability at the gene level that prevents primer annealing, though speculative. Regarding *P. prasina*, to our knowledge this is the first report showing the phylogenetic position of its *Pantoea* primary symbiont, as no sequences were available in the NCBI database. The closest match corresponded to a gammaproteobacterium identified as the symbiont of *Palomena angulosa* (*i.e.,* the closest relative of *P. prasina* for which a symbiont sequence was available) with a similarity of 99%. This result, together with the evidence that symbionts of *Palomena* spp. are in the same clade as those of *N. viridula* (which, like *P. prasina*, belongs to the tribe Nezarini) suggests a degree of co-evolutionary adaptation between *Pantoea* symbionts and at least some pentatomid hosts.

Besides providing confirmation of the identity of the main symbionts of the tested pentatomid species, an additional result was the retrieval of two 16S rRNA gene variants in *R. nebulosa*: variant 1 was identical to the sequence reported by Duron and Noël (26) and present in the majority of the samples evaluated; variant 2 showed slight divergence from variant 1 and was only present in two samples. The differentiated frequency of infection with different variants may be related to competition and consequent different transmission efficiency. In *Plautia stali* Scott, infection with multiple variants and symbiont replacement by environmental strains was reported (35); this possibility should be further explored for the stink bug species tested here. In contrast, no 16S rRNA gene variability was detected in symbionts of conspecific specimens of *G. italicum* and *D. baccarum*, confirming previous studies (26, 27). Interestingly, the occurrence of at least five symbiont variants was recently reported in the pentatomid *N. viridula*, and such variability was suggested to be related to a limited sensitivity to symbiont elimination (24). In the particular case of *N. viridula*, whose variable response to symbiont disruption is well recorded, the presence of different symbiont variants could explain this variability (23, 36). Whether symbiont variants compete for insect colonization or specific acquisition is a casual process is currently unknown; however, the adaptation to an irregular symbiont transfer pathway may also have supported an imperfect transmission, leading to a reduced sensitivity to symbiont suppression. Only a deeper study of sequence diversity in the tested pentatomids (*e.g.*, by high-throughput amplicon sequencing of the full-length 16S rRNA gene) could confirm the moderate frequency of symbiont variants. However, our approach allowed the identification of the dominant symbiont variant in each species, which is the one potentially most exposed to anti-symbiont treatment due to its large abundance.

The V4 midgut ventricle of pentatomids is usually colonized by one species identified as the primary symbiont of the host (36, 37). In this study, while targeting the symbiont residing the V4 ventricle, other bacteria were detected together with the primary *Pantoea* symbiont in all stink bug species except for *R. nebulosa* and *G. italicum*. Mainly, strains from the genus *Enterococcus* and *Serratia* were present in the samples. Bacteria from these genera are considered part of the non-transient microbiota in the southern green stink bug *N. viridula* that resides other parts of the midgut (37, 38). *Serratia* spp. (mainly *S. marcescens*) is a common entomopathogenic bacterium recorded in *P. prasina* midgut (39), in *N. viridula* nymphs (24) and other stink bugs such as *Piezodorus guildinii* (Westwood) (40). The sequences recorded in this study that were not assigned to the symbionts may come from the V2 and V3 ventricle rather than the V4 ventricle; their detection could therefore be owing to the dragging of microscopic parts of other regions during insect dissection. However, we cannot exclude that some of the recorded bacteria had colonized the V4 ventricle in the absence of *Pantoea* acquisition as a nymph, especially considering that our symbiont disruption experiments suggest a natural imperfect vertical transmission.

After egg masses treatment with an anti-symbiont biocomplex, a reduction in the hatching rate of *D. baccarum* was detected, as well as an increase in the same parameter for *G. italicum* and a reduction of the time required for egg hatching in *P. prasina.* This result contrasts with what observed in previous studies in the same species (26, 27). However, a complete egg sterilization was performed in those studies with bleach and ethanol and with formalin, respectively, followed by a washing step with water. In our case, the reductive effect on egg hatching observed for *D. baccarum* may be related to the tested biocomplex having an action on the egg development, perhaps by penetrating the chorion. Similar results were obtained when treating egg masses of *H. halys* using other compounds (22, 41, 42); however the mechanisms by which this occurs have not been clarified. The sensitivity of stink bug eggs to xenobiotics has been previously reported. For example, pre-emergence nymphal mortality was attributed to some foliar insecticides when applied on *N. viridula*’s egg masses (43). In contrast, both the anticipated egg hatching in *P. prasina* and the egg hatching increase in *G. italicum* after anti-symbiont treatment remain unclear. It may be supposed that the specific biological requirements of these species may let them benefit more than the others from a relatively more humid environment in the Petri dish due to the treatment with the biocomplex; alternatively a suppression of environmental pathogens from the egg surface may have occurred. Additional work would be needed to clarify the ovicidal effect of anti-symbiont treatment as well as the mechanisms regulating the specific response of *P. prasina* and *G. italicum* egg masses.

After egg hatching, we reported a variable effect on first instar nymphs according to the tested species. Considering that the experimental conditions did not change in any way during the experiments, the variation of the recorded effect must be attributed to a different response of nymphs according to the species. The first instar mortality of *C. purpureipennis* and *G. italicum* increased significantly after egg masses treatment with the anti-symbiont biocomplex. Similar studies performed on *G. italicum* showed no differences in mortality, although in that case the complete cycle of the insect was considered (not the first instar separately) and a different experimental set was used (26). Conversely, egg mass sterilization performed on *G. lineatum*, closely related to *G. italicum*, led to a longer life cycle and reduced fecundity in aposymbiotic individuals (28). For *D. baccarum* and *P. prasina*, no effect was observed on the mortality rates of the first instar. This result agrees with Itoh et al. (27), who did not observe significant differences in the first instar survival, although in that case the probability to survive to adulthood decreased notably in aposymbiotic insects.

The treatment with the anti-symbiont biocomplex reduced numerically the total number of nymphs that were symbiont-infected, according to PCR screening with specific primers. However, the tested biocomplex did not completely eliminate the symbionts. The lack of an absolute efficacy of the tested biocomplex was already observed when treating egg masses of *N. viridula* (24). Certainly, one of the challenges faced by symbiont-targeted control regards the use of products with variable anti-bacterial activity (42). On the other hand, many control nymphs were symbiont-negative. This pattern was observed as well in studies on *N. viridula* (20, 24) and could be attributed to an incomplete efficacy of symbiont acquisition by the nymphs. Such a naturally occurring imperfect symbiont transmission may have limited the insect dependence on their *Pantoea* affiliates, resulting in a lower impact of anti-symbiont treatment. Interestingly, the species for which no significant differences were detected in the mortality during the first instar (*D. baccarum* and *P. prasina*), were also those showing no significant differences in the symbiont presence in treated *vs* control nymphs, confirming an important relation between the manipulation of gut symbioses and mortality of newborns. The lack of a significant difference of *Pantoea* infection recorded for *D. baccarum* and *P. prasina* might be attributed to a reduced acquisition in the control nymphs as well, joint to a mild efficacy of the anti-symbiont biocomplex against their symbionts. A similar phenomenon has been reported for *N. viridula* and could be related to a transitory status of these symbiotic relationships from obligate to facultative. It would be worth further exploring the dependency level of the tested stink bugs on their symbionts, to improve knowledge on the suggested transition of gut symbioses in pentatomids.

On the whole, these experiments, by indicating variable response to symbiont disruption in pentatomid stink bugs, suggest that a large-scale application of symbiont-targeted pest control would not necessarily result in efficient containment of multiple pests. This means that symbiotic control measures applied against sensitive pests such as *H. halys* could not prevent at the same time from the emergence of secondary pests; nonetheless, considering the variability in insect response to symbiont removal, species-specific studies are needed to address each potential concerning stink bug. On the other hand, it is important to point out that a non-uniform insect suppression is not expected to produce a high impact on the biodiversity of agroecosystems. Many of the tested species are alternative hosts for the natural enemies of the main stink bugs pests (44–46), hence representing an important reservoir for the ecosystem services provided by them. Although extensive studies should be undertaken at the field or even landscape level to fully understand the long-time impact on the complex stink bug biocoenoses (*i.e.* by monitoring the abundance of key species over the years in treated areas), the preliminary evidence shown in this study supports the environmental sustainability of anti-symbiont pest control.

## Data Availability

The datasets presented in this study can be found in online repositories. The names of the repository/repositories and accession number(s) can be found below: NCBI, https://www.ncbi.nlm.nih.gov/, accession PP469563–PP469568, PP469574 and PP469575.
