# Supplementary material for: Effect of symbiont-targeted control of Halyomorpha halys on the co-occurring pentatomid community
Source: Front Insect Sci. 2025 Feb 5;5:1520065. doi: 10.3389/finsc.2025.1520065 (PMC11836026; doi:10.3389/finsc.2025.1520065)
Supplement: Supplementary file 1 [file Table1.docx]

Effect of symbiont-targeted control of Halyomorpha halys on the co-occurring pentatomid community

Supplementary Material

**Supplementary Table S1**. Details of collection localities of the tested stink bug species. Collection sites are indicated together with the Province: AL = Alessandria, TO = Torino.

| **Stink bug species** | **Collection site** | **Coordinates** | **Date of collection** | **Crop / host plant** |
| --- | --- | --- | --- | --- |
| *Carpocoris purpureipennis* | Pancalieri (TO) | 44°50'44.7"N, 7°36'18.8"E | May 2022 | Wheat |
| *Dolycoris baccarum* | Carmagnola (TO) | 44°52'33.8"N, 7°45'45.8"E | July 2022 | Soybean |
| *Graphosoma italicum* | Grugliasco (TO) | 45°03'59.3"N, 7°35'19.6"E | May 2022 | Wild vegetation (*Anthriscus sylvestris*) |
| *Palomena prasina* | Cereseto (AL) | 45°05'12.3"N, 8°19'17.1"E | June 2022 | Hazelnut |
| *Rhaphigaster nebulosa* | Candiolo (TO) | 44°57'48.5"N, 7°34'08.1"E | October 2022 | Wild vegetation (*Acer* spp.) |
